# Macroscale Robust Superlubricity on Metallic NbB_2_

**DOI:** 10.1002/advs.202103815

**Published:** 2022-03-10

**Authors:** Jia Wang, Chang Liu, Kaifei Miao, Kan Zhang, Weitao Zheng, Changfeng Chen

**Affiliations:** ^1^ State Key Laboratory of Superhard Materials Department of Materials Science and Key Laboratory of Automobile Materials MOE Jilin University Changchun 130012 China; ^2^ Department of Materials Science and Engineering Jilin Jianzhu University Changchun 130118 China; ^3^ International Center for Computational Methods and Software College of Physics Jilin University Changchun 130012 China; ^4^ Department of Physics and Astronomy University of Nevada, Las Vegas Las Vegas NV 89154 USA

**Keywords:** macroscale, metallic, NbB_2_, superlow wear, superlubricity

## Abstract

Robust superlubricity (RSL), defined by concurrent superlow friction and wear, holds great promise for reducing material and energy loss in vast industrial and technological operations. Despite recent advances, challenges remain in finding materials that exhibit RSL on macrolength and time scales and possess vigorous electrical conduction ability. Here, the discovery of RSL is reported on hydrated NbB_2_ films that exhibit vanishingly small coefficient of friction (0.001–0.006) and superlow wear rate (≈10^−17^ m^3^ N^−1^ m^−1^) on large length scales reaching millimeter range and prolonged time scales lasting through extensive loading durations. Moreover, the measured low resistivity (≈10^−6^ Ω m) of the synthesized NbB_2_ film indicates ample capability for electrical conduction, extending macroscale RSL to hitherto largely untapped metallic materials. Pertinent microscopic mechanisms are elucidated by deciphering the intricate load‐driven chemical reactions that generate and sustain the observed superlubricating state and assessing the strong stress responses under diverse strains that produce the superior durability.

## Graphical abstract

Here, the discovery of robust superlubricity on hydrated NbB_2_ film with superlow wear on macrolength and time scales is reported. The measured low resistivity of NbB_2_ indicates ample capability for electrical conduction, extending macro‐superlubricity to hitherto largely untapped metallic materials. These findings open fresh opportunities for implementing superlubricity in versatile applications that require electrical conduction as a prominent functionality.

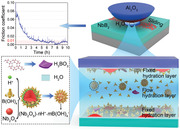

## 1 Introduction

The material and energy loss due to friction is a serious impediment on industrial and technological production and operation with negative impacts on the environment. Improving the efficiency and durability of moving mechanical parts via lubrication has been an enduring challenge to ancient technologies and modern science and engineering alike. The millennia‐old tradition of lubricating contacting surfaces with oil or grease continues to find broad applications in present‐day machineries; but developments in materials science have introduced more advanced techniques in treating surfaces via suitable coatings and using a variety of natural and synthetic solid and liquid lubricants. Recent years have seen the emergence and development of a distinct class of materials operating in a remarkable regime of motion known as superlubricity (SL), which is defined by nearly friction‐free (friction coefficient less than 0.01) states of motion.^[^ ^1^, ^2^ ^]^ Major efforts in SL research have focused on materials like graphite,^[^ ^3^, ^4^, ^5^ ^]^ graphene,^[^ ^6^, ^7^, ^8^ ^]^ hexagonal BN^[^ ^9^, ^10^ ^]^ and MoS_2_^[^ ^11^, ^12^, ^13^, ^14^ ^]^ that possess strong intralayer bonding but very weak interlayer interactions, allowing easy sliding motion with small friction, which is further diminished via specially tailored incommensurate interlayer structural alignments. Such so‐called structural SL is most viable at nano‐ to microscale where interlayer stacking can be effectively controlled. Extending this approach to larger length scales has faced formidable challenges,^[^ ^15^ ^]^ and elaborate structural designs have been proposed, such as diamond‐like carbon (DLC), nanodiamond and graphene,^[^ ^16^ ^]^ and MoS_2_ patches.^[^ ^17^ ^]^ Among notable developments in recent years are amorphous carbon films that exhibit SL behaviors under hydrogenation^[^ ^18^ ^]^ or lubrication with organic friction modifiers,^[^ ^19^ ^]^ while enhanced engineering designs have been proposed based on the combination of amorphous carbon film and additional carbon nanostructures.^[^ ^20^ ^]^

Another major approach in this research field invokes the concept of liquid‐based SL state, which does not suffer from many physical limitations associated with other SL mechanisms and can be more easily scaled up to macroscale.^[^ ^21^ ^]^ Of particular interest are water‐based lubricants,^[^ ^22^, ^23^ ^]^ which are more environment friendly than traditional oil‐based lubricants. Major examples include acid‐,^[^ ^24^ ^]^ ionic liquid‐,^[^ ^25^ ^]^ and nanomaterial‐based aqueous lubricants,^[^ ^26^, ^27^, ^28^ ^]^ which operate based on the principles of hydrodynamic lubrication of water, hydrogen‐bond network for viscous lubricants, and “stern layer” and hydrogen‐bond network for acid‐based lubricants.^[^ ^21^ ^]^ The use of additives, however, still poses major material and environmental challenges, such as severe wear, acidic conditions and stringent surface preparation requirements. These pressing issues call for cleaner water‐based lubrication, which hinges on finding materials that exhibit SL under simple hydration conditions. While significant researches have focused on carefully designing material combinations^[^ ^16^ ^]^ or artificially constructing SL environments,^[^ ^29^ ^]^ a pressing task is to explore new friction materials that can spontaneously form macro‐SL state in the ambient environment. Additionally, SL materials also need to possess low wear rate for high durability. Because of the high valence electron density and strong covalent bonds, transition‐metal diborides with excellent intrinsic mechanical properties, chemical inertness and oxidation resistance have great potential to provide strong wear resistance in water‐based environment.^[^ ^30^, ^31^, ^32^, ^33^, ^34^ ^]^ Many device applications further require the coating materials to conduct electricity, but metallic macroscale SL materials have been conspicuously lacking, leaving this prominent functional demand unattained.

In this article, we report an intriguing discovery of macroscale robust superlubricity (RSL) in metallic transition‐metal diboride NbB_2_. This outstanding regime of motion is characterized by concurrent superlow friction and wear with exceedingly small friction coefficient of 0.001–0.006 and vanishingly low wear rate around 10^−17^ m^3^ N^−1^ m^−1^. Notably, the synthesized NbB_2_ film has low measured resistivity on the order of 10^−6^ Ω m, which establishes macroscale RSL for the first time among metallic materials. The observed RSL is generated and sustained by the friction process on the hydrated specimen surface, which is viable in humid ambient environments without requiring chemical additives, thus facilitating practical implementations. We first performed tribological tests on the NbB_2_ film using the standard counterpart ball and then used a tailor‐made counterpart pin with a greatly enlarged contacting surface area, demonstrating RSL on length scales reaching the state‐of‐the‐art millimeter range for macroscale SL. We elucidate atomistic mechanisms for the observed phenomena by analyzing the key chemical reactions that generate the SL state and assessing the structural and stress responses that maintain the superior structural durability with minimal wear during the friction loading process.

The present findings extend macroscale RSL into the prominent class of metallic materials, enabling applications in diverse equipment and device settings where RSL has not yet been achieved. This work also lays a solid foundation for further exploration and development of additional metallic RSL materials among transition‐metal diborides and possibly other related compounds that are capable of operating in maintenance‐free and environment friendly conditions, opening paths for new versatile implementations of RSL in a broad variety and range of industrial and technological applications.

## 2 Results

### 2.1 Synthesis and Characterization of NbB_2_ Films

**Figure 1:**
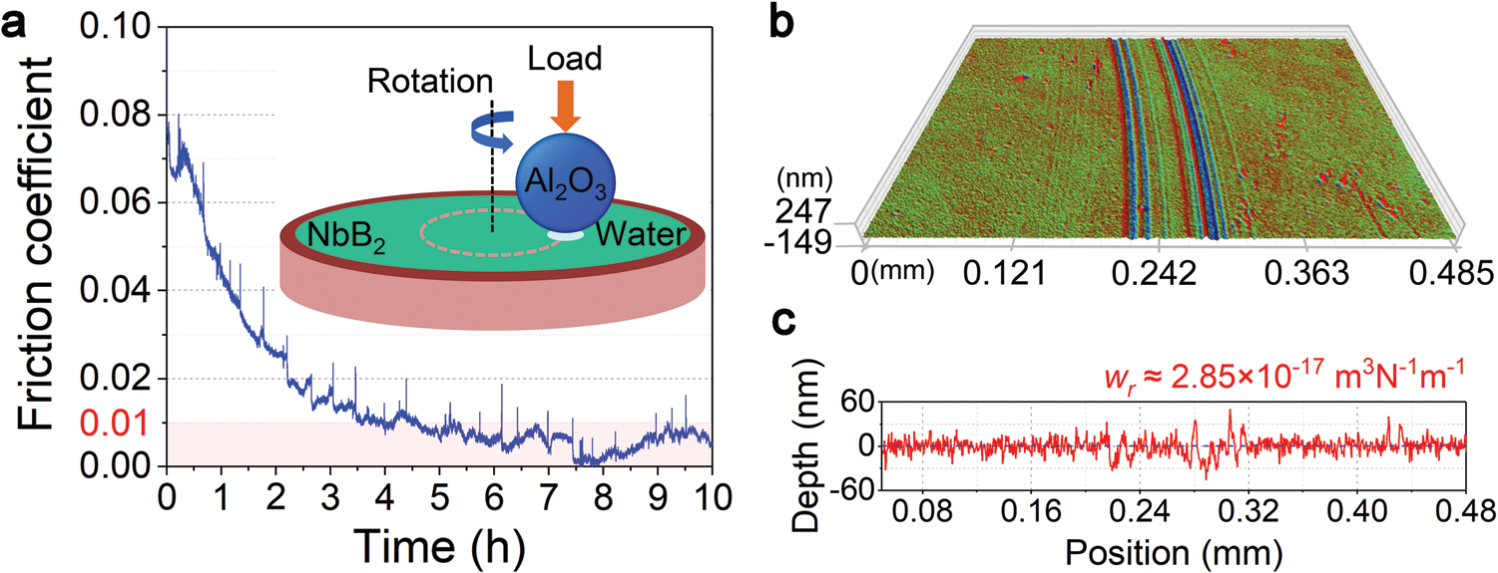
Tribological characterization of the NbB_2_ film in the ball‐on‐disk mode. a) Dynamic friction coefficient of the highly crystallized NbB_2_ film measured in the ball‐on‐disk mode. The inset is a schematic diagram of the ball‐on‐disk tribotest setup in the film–water–air three‐phase contact mode. After a break‐in period of about 3.5 h, the friction coefficient drops below 0.01, entering an SL state that lasts for the subsequent 6.5 h test duration with an average friction coefficient of 0.0075. No abatement of the SL state is seen to the end of the tribotest, indicating a long‐lasting load‐driven and sustained SL mechanism. b,c) A 3D profile image and a cross‐sectional view of the NbB_2_ film wear track taken after the 10 h tribotest. To measure the abrasion state, the film is ultrasound treated with ethanol before taking the 3D profile and cross‐sectional characterizations. The wear rate of 2.85 × 10^−17^ m^3^ N^−1^ m^−1^ for the NbB_2_ film is calculated by fitting the cross‐sectional data.

We synthesized NbB_2_ in thin film on Si substrate using the magnetron sputtering technique and obtained specimens in good crystallinity with the (001) texture in the simple hexagonal structure, the thickness of the NbB_2_ film is about 720 nm (Figure S1, Supporting Information). We then performed tribotests using a ball‐on‐disc tribometer as depicted in the inset of **Figure** **1**a (see the Experimental Section for more details). Deionized water droplets were added to the tribopair contact area to hydrate the film surface, resulting in a steady‐state water bridge connecting the film surface and the tribometer ball. This solid (tribopairs), liquid (water bridge), and gas (air) three‐phase contact mode is highly conducive to generating and sustaining RSL on NbB_2_ film (see below for details). Under the three‐phase contact, the friction coefficient of NbB_2_ film starts at 0.06–0.07 and declines steadily as the test progresses (Figure 1a); after a break‐in period of 3.5 h, the friction coefficient drops below 0.01, entering an SL state that lasts for the entire subsequent test period with an average value of 0.0075. The SL state stays robust for the remaining 6.5 h of the experimental run without any abatement to the end, indicating long‐lasting SL induced and maintained by load‐driven self‐sustaining lubricating mechanisms. In subsequent experiments, the repeatability of the SL state for the NbB_2_ film was further verified by tests under a range of loads and velocities (see Figure S2 in the Supporting Information).

For materials exhibiting SL behaviors, durability is also a prominent consideration for practical applications; a superlow wear rate is therefore another hallmark that is a highly desired and sought‐after quality for RSL materials. To assess this characteristic property, we have examined the wear track by taking optical microscope images after the tribotests, and the resulting images show no visible accumulation of wear debris (Figure S3, Supporting Information). After a thorough ultrasonic treatment with ethanol, the wear track was observed by a 3D optical profilometer, and the results (Figure 1b) show only slight and shallow abrasion. An analysis of the cross‐sectional depth (Figure 1c) yields a vanishingly low wear rate of 2.85 × 10^−17^ m^3^ N^−1^ m^−1^, which is among the smallest values for durable coating materials. This striking phenomenon is fundamentally rooted in the superior mechanical strength stemming from the strong bonding structure of NbB_2_, with measured nanoindentation hardness (*H*) reaching 38.8 ± 1.4 GPa (elastic modulus (*E*) is 367.6 ± 2.8 GPa), which underlies both the long break‐in period for the onset of SL behaviors and the high durability during frictional loading conditions. We will elucidate the atomistic mechanisms based on systematic first‐principles evaluations of the stress–strain relations of NbB_2_ later in this article.

To explore the effect of variable degrees of crystallinity on tribological properties, we have prepared NbB_2_ films in less crystallized forms and run additional tribotests on such NbB_2_ films. The results (Figure S4, Supporting Information) show that such films all enter similar SL states with dynamic friction coefficients dropping below 0.01. Interestingly, these less crystallized films require notably reduced amounts of break‐in time to enter the SL state, indicating that such structurally weakened NbB_2_ films are more conducive to releasing contents that promote the load‐driven lubricating mechanism, evincing that the tribo‐products from the NbB_2_ films play a major role in generating the SL states.

To assess the electrical conduction capacity of the synthesized NbB_2_ film, we have performed four‐probe resistance measurements (see the Experimental Section for details). The obtained resistivity of (7.44 ± 0.24) × 10^−7^ Ω m indicates good ability for electrical conduction of the NbB_2_ film. This result is consistent with the calculated electronic density of states data (see below) that reveal robust metallic nature of NbB_2_ both at equilibrium and at large loading strains. It is noted that macroscale SL materials reported to date have been dominated by strong covalent materials, operating in dry or liquid environment.^[^ ^21^ ^]^ The first category includes DLC/nanodiamond/graphene,^[^ ^16^ ^]^ DLC/nanodiamond/MoS_2_,^[^ ^17^ ^]^ ta‐C/ta‐C in unsaturated fatty acids or glycerol,^[^ ^19^ ^]^ and fullerene‐like MoS_2_/steel ball;^[^ ^11^ ^]^ the second category includes Si_3_N_4_/Si_3_N_4_ in water,^[^ ^35^ ^]^ Si_3_N_4_/SiC in water,^[^ ^36^ ^]^ CN*_x_*/SiC in water,^[^ ^37^ ^]^ Si_3_N_4_/SiO_2_ in water containing lubricating additives,^[^ ^26^, ^28^, ^38^ ^]^ and Si_3_N_4_/Si_3_N_4_ in ionic liquid.^[^ ^25^, ^39^ ^]^ These materials are all electrically nonconducting with fairly large electronic band gaps that are typical for strong covalent solids. In a recent study, SL was reported in electrically conductive 2D titanium carbide (Ti_3_C_2_) MXene coating sliding against DLC in a dry nitrogen protection environment.^[^ ^40^ ^]^ However, as a layered material similar to graphene, the lower mechanical hardness of MXene seriously impedes its service life.^[^ ^41^ ^]^ The presently reported NbB_2_ films with RSL characteristics represent a distinct type of materials that possesses the rare combination of superlow friction and wear rate with good electrical conducting capacity. This finding opens new possibilities for implementing RSL in more versatile applications where vigorous electrical conduction is required.

**Figure 2:**
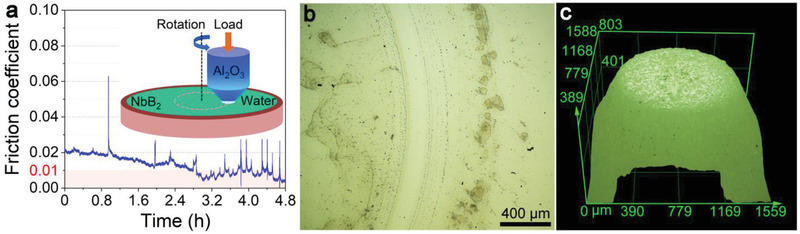
Tribological characterization of the NbB_2_ film in the pin‐on‐disk mode. a) Dynamic friction coefficient of the highly crystallized NbB_2_ film measured in the pin‐on‐disk mode. The inset is a schematic diagram of the pin‐on‐disk tribotest setup in the three‐phase contact mode. The Al_2_O_3_ counterpart pin has a diameter of 1 mm. Superlubricity occurs after a 2.8 h break‐in period and remains unabated for the rest of the tribotest period with an average friction coefficient of 0.0082. Also shown are the optical microscope images of b) the NbB_2_ film wear track and c) the counterpart pin wear scar taken without any preprocessing.

The RSL of NbB_2_ requires no special structural alignment or maintenance at the friction surfaces and, therefore, is expected to scale up readily to large length scales. To verify this idea, we replaced the standard Al_2_O_3_ counterpart ball, which operates in a point‐contact mode, with a tailor‐made Al_2_O_3_ pin that has a circular contacting surface of a 1.00 mm diameter, as shown in **Figure** **2**a. During the tribotests, the dynamic friction coefficient initially at about 0.02 continues to decline and SL sets in after a 2.8 h break‐in period and lasts for the rest of duration of the test with an average friction coefficient of 0.0082. The optical microscope images shown in Figure 2b,c reveal that the wear track and the pin contact scar show no obvious wear except for some tribo‐products. This result shows that the NbB_2_ film in the three‐phase contact environment can reach the RSL state on the millimeter length scale in a large surface‐contact mode.

The present results are expected to open fresh avenues for discovering more RSL materials among metallic compounds, but further advances hinge on a comprehensive understanding of fundamental mechanisms underlying the experimentally observed phenomena. To facilitate this process, we have performed a systematic examination of the tribological procedure and products to analyze the associated atomistic processes. Our analysis reveals key chemical, structural and mechanical properties that underpin the superior tribological characters of the NbB_2_ films, which are elaborated below.

### 2.2 Mechanism for Superlubricity of Hydrated NbB_2_

**Figure 3:**
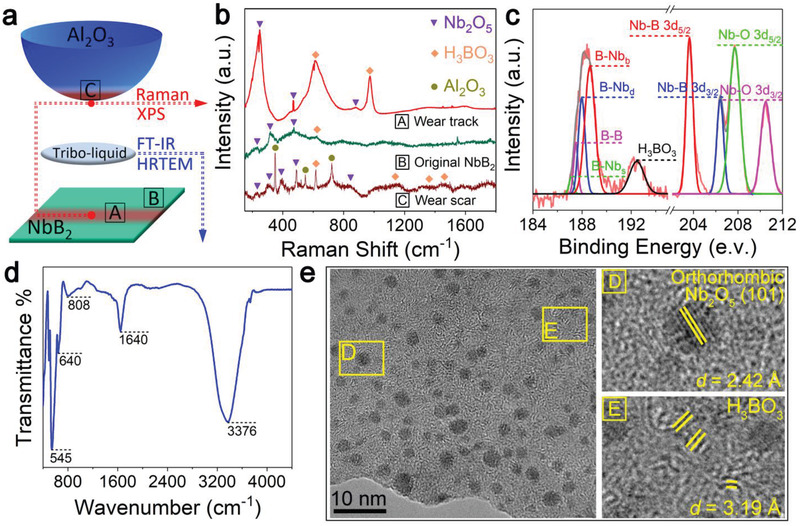
Post‐tribotest characterization of the NbB_2_ film wear track, counterpart ball wear scar and tribo‐liquid. a) Schematic diagram of the experimental procedure. After the tribotest, the wear track and wear scar are characterized directly, and the “tribo‐liquid” between the friction surfaces is extracted by pipette for subsequent characterizations. “A” represents the center position of wear track, “B” represents the original NbB_2_ film, and “C” represents the center position of wear scar. b) Raman spectra of wear track, original NbB_2_ film, and wear scar. In addition to the peaks from spontaneous oxidation on the NbB_2_ film surface, more peaks of H_3_BO_3_ and Nb_2_O_5_ with higher intensity appeared on the tribopairs, with the peaks of Al_2_O_3_ (350, 548, and 720 cm^−1^)^[^ ^42^ ^]^ on wear scar originating from the counterpart ball itself. c) XPS Nb 3d and B 1s spectra of the NbB_2_ film wear track. d) FT‐IR spectrum of the tribo‐liquid collected after the tribotest. H_3_BO_3_ and Nb_2_O_5_ are the main tribo‐products dissolved in the water droplet. e) HRTEM images of the particles in the tribo‐liquid. The amorphous H_3_BO_3_ matrix contains a large number of well dispersed Nb_2_O_5_ nanoparticles.

To elucidate atomistic mechanisms for the SL state of hydrated NbB_2_, we have performed a thorough analysis of the friction surfaces following the tribotests. We have carried out Raman characterization on two distinct areas, one away and another at the center of the wear track. In the first case, weak H_3_BO_3_ (620 cm^−1^)^[^ ^43^ ^]^ and Nb_2_O_5_ (235, 314, and 470 cm^−1^)^[^ ^44^, ^45^ ^]^ peaks are detected, as shown in **Figure** **3**b, indicating oxidation of the film surface that is exposed to ambient air. In contrast, a notably stronger Nb_2_O_5_ peak (250 cm^−1^)^[^ ^46^ ^]^ accompanied by two weak Nb_2_O_5_ peaks (470 and 880 cm^−1^)^[^ ^44^, ^45^ ^]^ appear in the spectra taken at the wear track center, indicating that a large amount of Nb_2_O_5_ has formed during the tribotests. Additionally, two very strong H_3_BO_3_ peaks (610 and 975 cm^−1^)^[^ ^43^, ^47^ ^]^ are detected, indicating that H_3_BO_3_ is among the main oxidative products generated by the friction process.

To corroborate the above bonding assignments based on the Raman spectra, we have further performed X‐ray photoelectron spectroscopy (XPS) measurements of the wear track. The results (Figure 3c) show that in addition to the spectral features attributed to the bonds in the original NbB_2_ film, the B 1s and Nb 3d spectra reveal B—O bonds in H_3_BO_3_ and Nb—O 3d_5/2_ and Nb—O 3d_3/2_ bonds in Nb_2_O_5_. Moreover, we examined the tribo‐products collected from the wear scar center of the counterpart ball and observed the Raman peaks of H_3_BO_3_ (617, 1105, 1365, and 1449 cm^−1^)^[^ ^47^, ^48^ ^]^ and Nb_2_O_5_ (235, 306, 390, 485, and 845 cm^−1^)^[^ ^44^, ^45^ ^]^ (Figure 3b). These results offer compelling evidence on substantial friction generated H_3_BO_3_ and Nb_2_O_5_, which help promote the SL state at the surface of the NbB_2_ film. Meanwhile, tribotests in dry or water‐covered environments also produce H_3_BO_3_ and Nb_2_O_5_ yet fail to produce SL states (Figure S5, Supporting Information), indicating that additional conditions need to be met to induce superlubricity.

Friction induced tribo‐products dissolve into the “water bridge” to produce a certain concentration of charged colloids via the Tyndall effect (Figure S6, Supporting Information). The average zeta potential of the “water bridge” content collected after the tribotests is about −8.45 mV, clearly indicating that it is negatively charged (Figure S7, Supporting Information). The Fourier‐transform infrared (FTIR) spectrum of the tribo‐liquid (Figure 3d) reveals that besides the peak associated with the O–H stretching (3376 cm^−1^), the distinct B–O peaks stemming from bending, out‐of‐plane bending and symmetric stretching (545, 640, and 808 cm^−1^) and a signal peak related to Nb_2_O_5_ (1640 cm^−1^) are observed.^[^ ^43^, ^49^, ^50^ ^]^ High‐resolution transmission electron microscopy (HRTEM) measurements (Figure 3e) show small particles well dispersed in an amorphous matrix without aggregation. These dispersed particles comprise crystalline Nb_2_O_5_ grains, which are well matched with the (101) oriented orthorhombic‐Nb_2_O_5_ crystallites. Averaging results from more than 100 particles, the size distribution is ≈2.5 nm on average and 2.25–3 nm in range with the measurement result of 2.25 nm accounting for nearly 50% of the particles. It is noted that the identified amorphous matrix is not completely disordered but rather displays small curved layers with fingerprint‐like structures. Furthermore, the interlamellar spacing of the short‐range ordered layer with fingerprint‐like structures is about 3.19 Å, which is well matched with the structural feature of H_3_BO_3_, so the characteristic fingerprint‐like structures likely stem from the formation of H_3_BO_3_ lamellae after the evaporation of water. These results identify H_3_BO_3_ and Nb_2_O_5_ colloidal particles as key agents that work synergistically at the friction surfaces to achieve superlubricity.

**Figure 4:**
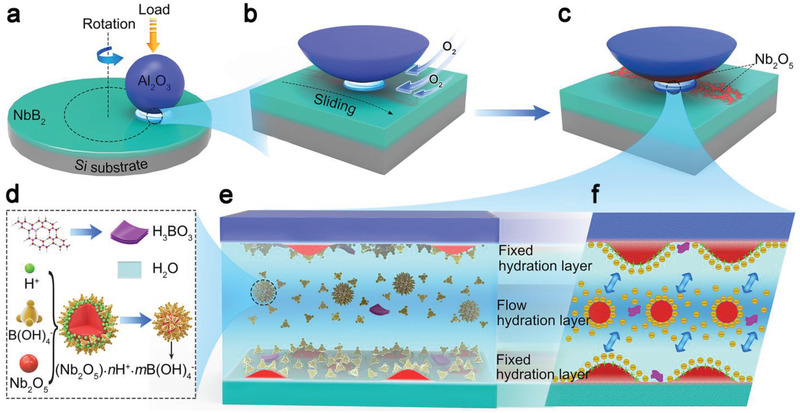
Schematics of the model and mechanisms for superlubricating hydrated NbB_2_ film. Illustration of a) the ball‐on‐disk loading setup for the tribotest on the hydrated NbB_2_ film, b) the frictional sliding process in the ambient air that supplies the oxygen needed for the tribochemical reactions, and c) the resultant Nb_2_O_5_ and d) other species that promote a series of reactions including oxidation, ionization, adsorption, and complexation to form the tribo‐products ([Nb_2_O_5_]·*n*H^+^·*m*B[OH]_4_^−^ and B[OH]_4_^−^), which lead to the formation of e) two fixed hydration layers and a flowing hydration layer between the friction surfaces, where f) the ionic repulsion helps to reduce the contact pressure and the associated friction, thereby facilitating realization of the SL state.

We now present an in‐depth analysis of the three‐phase contact environment at the surface of NbB_2_. As depicted in **Figure** **4**a–c, promoted by friction, the wear surface of NbB_2_ undergoes an oxidation reaction with O_2_ in the air, generating B_2_O_3_ and Nb_2_O_5_, and the intermediate oxidative product (B_2_O_3_) further reacts with H_2_O to form H_3_BO_3_. The total reaction process is described by

(1) $$4\mathrm{Nb}B_{2}+12H_{2}O+11O_{2}\to 8H_{3}BO_{3}+2Nb_{2}O_{5}$$

The resulting tribo‐products dissolve in the “water bridge,” producing a tribo‐liquid containing a large amount of H_3_BO_3_ and Nb_2_O_5_ in the 4:1 ratio. As a typical monobasic weak acid, the H_3_BO_3_ easily ionizes in water, generating tetrahydroxy borate ions in the B‐centered tetra‐coordinate units (B(OH)_4_^−^) and hydrogen ion (H^+^) via the reaction

(2) $$B{\left(\mathrm{HO}\right)}_{3}+H_{2}O\to B{\left(\mathrm{OH}\right)}_{4}^{-}+H^{+}$$

The ionization of H_3_BO_3_ acts as an ongoing source of H^+^, which is absorbed by Nb_2_O_5_ to form positively charged colloidal particles (Nb_2_O_5_)·*n*H^+^ via reaction

(3) $$Nb_{2}O_{5}+nH^{+}\to \left(Nb_{2}O_{5}\right)\cdot nH^{+}$$

further promoting the forward ionization reaction. The resulting tribo‐liquid contains ample B(OH)_4_^−^, (Nb_2_O_5_)·*n*H^+^ and H_3_BO_3_ generated by these tribochemical reactions.

Our post‐tribotest characterization has uncovered strong evidence of ample Nb_2_O_5_ and H_3_BO_3_ on the NbB_2_ surface. During friction, the three reactions described above reach a dynamic equilibrium. From Equations (1) and (2) and the conservation of reaction, the content of B(OH)_4_^−^ is much higher than that of (Nb_2_O_5_)·*n*H^+^. At the friction surface, positively charged (Nb_2_O_5_)·*n*H^+^ ions interact with negatively charged B(OH)_4_^−^ ions to form (Nb_2_O_5_)·*n*H^+^·*m*B(OH)_4_^−^ via

(4) $$\left(Nb_{2}O_{5}\right)\cdot nH^{+}+mB{\left(\mathrm{OH}\right)}_{4}^{-}\to \left(Nb_{2}O_{5}\right)\cdot nH^{+}\cdot mB{\left(\mathrm{OH}\right)}_{4}^{-}$$

The depiction of Equation (4) is shown in Figure 4d. Size effects of B(OH)_4_^−^ prevent proportional reaction, so *m* is less than *n* in the above equation. Therefore, stable tribo‐liquids at the friction surface contain (Nb_2_O_5_)·*n*H^+^·*m*B(OH)_4_^−^ and a large number of free B(OH)_4_^−^ (Figure 4e). These ingredients bond with water molecules through the hydrogen bonding, and the B(OH)_4_^−^ ions help neutralize (Nb_2_O_5_)·*n*H^+^ ions at the friction surface. Moreover, the tribo‐products on tribopair surfaces can fix a portion of water molecules to form a hydration layer^[^ ^51^ ^]^ that coexists with a flowing hydration layer containing tribo‐products between the upper and lower fixed hydration layers, as shown in Figure 4f. Overall, the number of negatively charged surface sites is greater than that of positively charged sites, making both friction surfaces net negatively charged. Meanwhile, the flowing hydration layer contains ample B(OH)_4_^−^ ions, leading to an electrical double‐layer repulsion between the flowing hydration layer and the two fixed hydration layers, reducing the contact pressure. Consequently, the flowing hydration layer exhibits its natural weak shear characteristics to produce the observed small friction.^[^ ^22^, ^52^ ^]^ According to further calculations by Hamrock‐Dowson theory,^[^ ^52^ ^]^ the lubrication regime of NbB_2_ film in stable SL state is located in hydrodynamic lubrication (Figure S8, Supporting Information), which is the result of the synergistic effect of three hydration layers and is directly affected by external friction conditions (Figure S2, Supporting Information). It is easy to achieve SL when the load does not exceed 2.5 N, and the probability of SL decreases as the load increases. Under higher loads, the subdued hydration layer cannot support the high contact pressure from the upper counterpart ball, which results in direct solid‐solid contact of the tribopair surfaces, thereby suppressing the SL state. Moreover, the SL state is realized at relatively low velocities (≤1.2 cm s^−1^). In hydrodynamic lubrication regime, the tribopairs are separated by the hydration layers sliding past each other, the shear stress (shear or friction force per unit surface area, *σ*) is directly proportional to the viscosity of the hydration layer (*η*) and the sliding velocity (*v*), and inversely proportional to the thickness of the hydration layer (*D*) via the relation *σ* = *ηv*/*D*, which indicates that shear stress varies linearly with sliding velocity.^[^ ^53^ ^]^ Under high velocity, the resistance to motion arises as the hydration layer is sheared, resulting in the suppression of the SL state. Due to the limitations of the tribotester, we did not investigate the influence of temperature on the SL state. However, according to the existing literature,^[^ ^54^, ^55^ ^]^ increasing temperature will weaken the adsorption capacity of colloidal particles and accelerate their movement, causing an increase of the collision chance between colloidal particles and resulting in the coagulation of colloidal particles, which destroy the interface repulsion and the stability of hydration layer. In addition, rising temperature will reduce the viscosity of tribo‐liquid and then affect the thickness of hydration layer (Figure S8b, Supporting Information), and also accelerate the evaporation of water to cause excessive moisture loss, resulting in destruction of the hydration layer. Overall, two factors are key to generating SL at the NbB_2_ surface: i) tribo‐products displaying hydrophilic properties to enable the formation of fixed hydration layers on the friction surface and ii) charged agents from tribochemical reactions generating interface and hydration layer repulsion to unleash the weak shear character of the tribo‐liquid. To verify the SL mechanism, we further studied the influence of the elemental composition of the film on achieving SL in the three‐phase contact environment. A niobium nitride (NbN) film was prepared as a contrasting material for tribotests in the three‐phase contact environment (Figure S9, Supporting Information). The results show that SL was not realized on the NbN film, without B(OH)_4_^−^ ions formed in water to generate effective interfacial repulsion as achieved in the case of the NbB_2_ film. In addition, we built a film–water–argon three‐phase contact environment to test the tribological behavior of the NbB_2_ film. Our results show that without the oxygen provided by the ambient air, the formation of H_3_BO_3_ and Nb_2_O_5_ is difficult and the NbB_2_ film cannot enter the SL state (Figure S10, Supporting Information).

### 2.3 Mechanism for the Superlow Wear Rate of NbB_2_

Transition‐metal borides possess very high mechanical strength stemming from their superior bonding structure, with the transition‐metal atoms provide a high count of valence electrons to resist compression and boron atoms form a strong covalent network to sustain large shear deformation.^[^ ^56^ ^]^ To elucidate the atomistic mechanisms for the superlow wear rate of NbB_2_, we have performed quantum mechanical calculations to examine the stress responses of NbB_2_ under the friction induced biaxial stress that comprises a shear stress component aligned in the frictional contact surface and a compressive stress normal to the contact surface. We have performed systematic calculations of stress–strain relations to assess pertinent mechanical properties (see the Experimental Section for computational details). This approach has been broadly employed in the study of many transition‐metal compounds, including a wide range of borides, and the results have provided accurate descriptions of a variety of structural and mechanical properties^[^ ^57^, ^58^, ^59^, ^60^, ^61^ ^]^ and proved to be a powerful tool in probing and elucidating prominent structure–property relations of wide‐ranging materials under versatile loading conditions.^[^ ^62^, ^63^, ^64^, ^65^ ^]^

**Figure 5:**
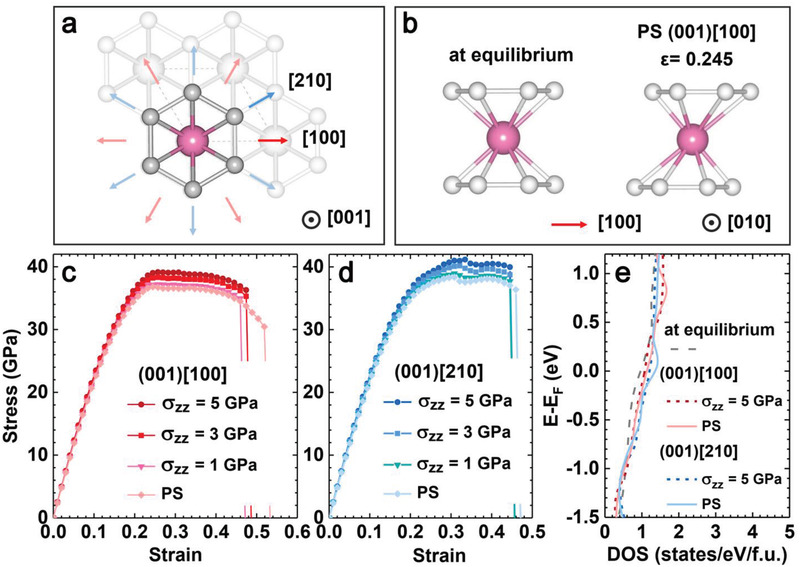
Atomistic mechanism for the superlow wear rate and metallicity of the NbB_2_ film. a) The crystal structure of NbB_2_ at equilibrium exhibiting sixfold rotational symmetry in the (001) plane, with the large and small balls representing the Nb and B atoms, respectively. The two major high‐symmetry directions, [100] and [210], are indicated by the heavy‐shaded arrows, along with the respective five additional equivalent directions for each case shown by the light‐shaded arrows in the same colors. b) The bonding pattern of the NbB_2_ structural unit under the pure shear strains along the (001)[100] direction at the peak stress compared with the unstrained equilibrium structure. c,d) The stress responses to the pure shear (PS) and biaxial (with an additional normal stress *σ_zz_* that is set at several values) strains in the (001)[100] and (001)[210] shear‐slip direction as indicated. e) Calculated electronic density of states of the NbB_2_ crystal that is at the unstrained equilibrium structure, at the peak stress points under the (001)[100] and (001)[210] pure shear strains, or at the peak stress points under the corresponding biaxial strains with the normal stress *σ_zz_* = 5 GPa.

To assess the atomistic mechanisms underlying the superlow wear rate of NbB_2_ observed in our tribotests, we have examined stress responses to frictional shear strains applied to the (001) oriented NbB_2_ specimen, which is the structural configuration of the synthesized and tested specimen (see **Figure** **5**a for the bonding structure in the (001) oriented NbB_2_). Our calculations first establish the stress responses under the pure shear strains along the high‐symmetry (001)[100] and (001)[210] slip directions to set the key benchmarks on the strength and toughness. The calculated results (see Figure 5c,d) reveal strong stress responses to the pure shear deformations, with the peak shear stresses reaching 37–38 GPa in the two major slip directions. The bonding structure in the NbB_2_ (001) plane has a sixfold rotational symmetry, which produces a densely packed and evenly spanned 12‐fold shear‐slip directions, as indicated by the two sets of colored arrows in Figure 5a, that make the (001) oriented NbB_2_ specimen strongly resistant to shear deformations. Moreover, after the initial steep rise, the stresses remain high and form extended plateaus over large strain ranges in both shear slip directions, which further enhances the strong and tough nature of the NbB_2_ crystal under these loading conditions. These remarkable mechanical characteristics can be attributed to the mixed metallic and covalent nature of the bonds in NbB_2_ that play prominent roles in determining the strength and toughness of the crystal. To elucidate the pertinent underlying atomistic mechanism, we have analyzed the bonding changes in response to the applied shear strains. We present in Figure 5b the bonding configuration of NbB_2_ at the peak stress under the (001)[100] pure shear strains compared with the bonding structure at equilibrium, and the results show that the mixed covalent‐metallic Nb—B bonds tilted in the shear‐slip direction undergo the most elongation and act as the major load‐bearing bonds under the shear loading. Similar changes occur consistently in bonding configurations with the Nb—B bonds tilted in the shear‐slip direction as the major load bearers under all the pure shear and biaxial strains examined in this work. These results explain the simultaneously strong and tough stress responses under diverse loading conditions in all the cases shown in Figure 5c,d.

Under the frictional loading conditions during the tribotests, the (001) oriented NbB_2_ crystal is subjected to the coexisting shear and normal stresses in a flat‐contact geometry. Accordingly, we have further calculated the shear stress responses in the presence of a flat‐contact normal stress that is omnipresent in such contact modes to assess the structural deformation modes and the associated mechanical strength that determine the durability of the synthesized NbB_2_ specimen. The obtained results (see Figure 5c,d) reveal consistently enhanced shear stresses at rising normal stresses, showcasing notable strain stiffening of NbB_2_ under the frictional contact environments. This load induced strengthening phenomenon is extraordinary among transition‐metal compounds, many of which are known to suffer the opposite effect, namely strain softening.^[^ ^57^, ^58^, ^59^, ^60^ ^]^ Moreover, the extended plateaus seen in the pure‐shear stress curves, indicating high toughness, remain robust under the normal‐stress constrained shear strains of frictional loadings. This behavior underscores the concurrent superior mechanical strength and toughness of NbB_2_ under the tribotest loadings, offering key insights into the atomistic mechanism for the exceptional durability as manifested in its superlow wear rate.

Furthermore, as valuable indicators for evaluating the near‐surface material properties in tribological applications, the ratios of *H*/*E* (related to the elastic strain to failure) and *H* ^3^/*E* ^2^ (related to the resistance to plastic deformation) have been shown to be the suitable parameters for predicting wear resistance.^[^ ^66^, ^67^ ^]^ From the nanoindentation measurement and subsequent calculations, *H*/*E* and *H* ^3^/*E* ^2^ for the NbB_2_ film are 0.106 and 0.43, respectively, which indicate the material studied here can be considered to be simultaneously hard and tough, providing reliable wear resistance.^[^ ^68^, ^69^, ^70^ ^]^ The intrinsic mechanical properties with high hardness, high mechanical strength and high toughness form the core basis that supports the NbB_2_ film to achieve the superlow wear in the three‐phase contact environment. Moreover, the interaction between the NbB_2_ film and friction environment also plays a role in influencing the film wear resistance. As a typical transition‐metal diborides, NbB_2_ shows the characteristics of strong chemical inertness, oxidation resistance and thermodynamic stability.^[^ ^71^, ^72^ ^]^ In ordinary atmospheric conditions, it is difficult to accelerate the oxidation process of NbB_2_ only by changing the water content of the surrounding environment. Due to the existence of the strong covalent bonds in NbB_2_, when oxygen in the environment diffuses into NbB_2_ bulk, there is a very high energy cost for B/O exchange to remove B, hindering further oxidation. This feature ensures that NbB_2_ will not be consumed too much to undergo oxidation in the three‐phase contact environment. Only during the friction process, the tribochemical reactions take place on the outermost layer of the NbB_2_ film. In the three‐phase contact environment, the gradually formed interface repulsion greatly reduces the wear probability caused by direct contact between the friction interfaces. With the formation and stabilization of the SL state, the process of tribochemical reactions of NbB_2_ slow down and become stable, which keeps the superlow wear of NbB_2_ film in the subsequent SL process. Overall, the superlow wear of the NbB_2_ film is jointly determined by its own mechanical properties, chemical inertness, and the interface repulsion formed during the friction process, while the intrinsic mechanical properties are the core material basis.

### 2.4 Electronic Density of States of Metallic NbB_2_ under Diverse Strains

To evaluate the capability for electrical conduction of the synthesized specimen under the various pure shear and biaxial frictional strains in comparison with the result of the unstrained crystal, we have calculated the electronic density of states of NbB_2_ under the related loading conditions. We present in Figure 5e the electronic density of states near the Fermi level under several representative loading conditions, including NbB_2_ crystal at unstrained equilibrium structure, deformed at the peak pure‐shear stresses along the (001)[100] and (001)[210] shear slip directions, and under the biaxial strains with the normal stress *σ_zz_* = 5 GPa along the two selected shear slip directions. These results show that the electronic states around the Fermi level of the deformed crystal remain rigorous and increase slightly, indicating that the good electrical conduction ability of the synthesized NbB_2_ specimen in the unstrained equilibrium configuration determined by the four‐probe resistance measurements presented above will remain robust under versatile loading conditions and persist to large strains encompassing the full elastic ranges. This result bodes well for meeting the requirement of consistently good electrical conduction in many device applications under diverse operational environments.

## 3 Discussion

The present work demonstrates macroscale robust superlubricity of metallic NbB_2_, which may be extended to other transition‐metal diborides that share the same material characters for achieving similar tribological performance with the exceptionally low friction and wear characters. Further exploration of these compounds is expected to produce better understanding of the effects of different chemical composition involving different transition‐metal atoms on the efficacy of the resulting tribo‐liquid for reducing friction at the three‐phase contact; another aspect of interest is a systematic examination of the effects of different transition metals on the structural and mechanical responses to friction loadings, which hold key to producing the superlow wear rate. We are hopeful that the present findings will stimulate further investigations of these key issues that may help find additional transition‐metal diborides that exhibit RSL behaviors.

The RSL of NbB_2_ is obtained under hydrated conditions characterized by a “water bridge” configuration formed at the dynamic friction surfaces in ambient environment. The combination of solid NbB_2_, tribo‐liquid, and ambient air comprises a three‐phase contact state that promotes tribochemical reactions producing H_3_BO_3_ and Nb_2_O_5_, which are continuously generated and dispersed into the “water bridge” for the formation of ionic (Nb_2_O_5_)·*n*H^+^·*m*B(OH)_4_^−^ and B(OH)_4_^−^, which are the crucial components of the tribo‐liquid generating and sustaining RSL. This load‐generated and self‐sustaining mechanism is highly robust and conducive to RSL on large length and time scales, as demonstrated in our experiments, making NbB_2_ and possibly additional transition‐metal diborides suitable for practical implementation in diverse applications.

Achieving the RSL state on NbB_2_ requires adequate surface hydration, because the tribochemical reactions for oxidation, ionization, adsorption, and complexation depend on properly supplied water. Otherwise, those reactions will proceed in reverse, and the tribological behaviors will display the characteristics of dry friction. In tribotests presented above, water droplets were added to the contacting interface between the tribopairs to facilitate the process. To verify the feasibility of truly self‐sustainable RSL, we have set up a controlled environment where a humidifier was used to generate humid air in a CSM tribotest chamber enclosed by a cover to retain the moisture, replacing dripping water as the source for hydrating the surface of NbB_2_ specimen. When the humidity inside the chamber rises to about 93%, gathering moisture at the interface of the tribopairs gradually condense and form a “water bridge.” The friction coefficient of the NbB_2_ film decreases with the increase of humidity, and the SL state is obtained after the three‐phase contact mode between the tribopairs is formed in the 93.4% high humidity environment (Figure S11, Supporting Information). This finding indicates that high environmental humidity may be a viable source for the three‐phase contact formation at the sliding contact. Such a “water bridge” also should be able to form in lower humidity environments over longer time period to allow sufficient water condensation at the tribopair interface. These observations and inferences bode well for implementing RSL in diverse controlled or natural humid environments.

This work establishes NbB_2_ as a distinct RSL material with concurrent possession of superlow friction, superlow wear and good electrical conducting capacity, thereby offering a highly desirable combination of performance characteristics that are needed in wide‐ranging device and equipment settings. An in‐depth analysis unveils the friction‐driven chemical reactions at the hydrated NbB_2_ surface in ambient air and the superior strengths under the frictional loading conditions stemming from the intrinsic strong bonding characters of NbB_2_ crystal as key microscopic mechanisms for the observed phenomena. These fundamental physics and chemistry insights may guide rational exploration and discovery of additional transition‐metal diborides and related compounds that exhibit metallic RSL behaviors on the macroscale. We expect that the present study will stimulate considerable interest and efforts for further research leading to vigorous development and implementation of RSL in versatile applications.

## 4 Experimental Section

### Synthesis of the NbB_2_ Film

NbB_2_ films were deposited on mirror‐polished n‐type Si (100) wafers by magnetron sputtering NbB_2_ target in a high‐vacuum system with a base pressure of 5 × 10^−4^ Pa. The Si substrates were ultrasonically cleaned in subsequent baths of acetone, ethanol and deionized water for 20 min, and then blown dry with N_2_ before deposition. During deposition, the working pressure was maintained around 0.7 Pa via controlling the flow rate of Ar at 80 sccm; the sputtering power on the NbB_2_ target was kept constant at 90 W (sputtering current at ≈0.3 A) by a DC power supply. Additionally, a −80 V bias voltage and a temperature of 400 °C were applied on the substrates during deposition. The target‐substrate distance and substrate rotational speed were fixed at 80 mm and 10 r min^−1^, respectively. An interlayer (Nb/NbN) was first deposited to enhance the film‐substrate adhesion.

### Structure and Morphology Characterization

By using a D8‐tools Bragg‐Brentano diffractometer with a Cu‐K*α* line (*λ* = 1.54056 Å), X‐ray diffraction (XRD) in the *θ*–2*θ* mode was conducted and the structural properties (such as interplanar spacing, crystal orientation, and grain size) of NbB_2_ were measured. The microstructure was obtained by HRTEM using a field‐emission JEOL 2010F microscope operated at 200 kV; the sheet specimen for the HRTEM measurement was prepared by mechanically scratching the NbB_2_ surface with a clean stainless‐steel blade. Analyses of composition and chemical states were carried out via PerkinElmer PHI‐5702 XPS; before measurement, 15 min Ar^+^ etching (1 keV etching energy) was carried out to remove any surface contaminants. Subsequently, the sectional measurements of NbB_2_ were conducted via SU 8010 scanning electron microscope (SEM) to explore the film growth condition. The corresponding surface morphology and root‐mean‐square roughness (*R*_q_) were obtained from using an atomic force microscope (AFM, Dimension Icon, Veeco Instruments, Bruker, Germany). The thickness of film was measured by Veeco Dektak 150 surface profiler. In addition, to determine the hardness and elasticity modulus of NbB_2_, nanoindentation measurement was performed with a penetration depth of 500 nm by MTS Nanoindenter XP with a 3‐side pyramidal Berkovich‐type diamond indenter.

### Tribotests and Postfriction Characterizations

Tribotests of NbB_2_ were performed using a CSM ball‐on‐disk macroscale tribometer along a circular track of 5 mm diameter against the Al_2_O_3_ ball (diameter of 6 mm and Rockwell Hardness of 80). During the tribotests, the normal load was kept at 1 N and the sliding speed at 1.0 cm s^−1^. Prior to the start of the tests, a pipette was used to drip about 5 µL of deionized water on the film surface to construct the three‐phase contact environment between the friction surfaces. Besides, friction tests in normal dry atmospheric environment and in a full water‐covered environment at room temperature were also performed to assess their influence on the tribological behaviors of NbB_2_. Zero calibration of the machine was performed automatically at the beginning of each test. After the tribotests, ex situ characterization and analysis of the worn surface were performed using a 3D optical profilometer (Bruker ContourGT). The wear rate was calculated from the volume of the removed material by measuring the cross‐sectional area at five different locations along the wear track. Further identifications were carried out to recognize the chemical composition of the wear track and wear scar by Raman spectroscopy (Renishaw 1000, 514.5 nm laser) with an output power of 2 W, and to analyze the relative content of the oxidation products by XPS carried out at the wear track after the tribotests. Also, the information about the surface of the wear track and wear scar was acquired by optical digital microscope (OLLYMPUS DSX510). The “tribo‐liquid” formed during the friction process was collected by a pipette for characterization via irradiation by infrared light to detect the Tyndall effect. The FT‐IR spectrum of “tribo‐liquid” was obtained on an ALPHA Fourier Transform Infrared Spectrometer (Bruker) with the ATR mode. Dropping the “tribo‐liquid” on Cu grids and letting water evaporate completely, the microstructures of colloidal particles in the “tribo‐liquid” were observed by HRTEM. The Zeta potential measurement of the “tribo‐liquid” was performed by using a nanoparticle potentiometer (Zetasizer Nano ZS). The viscosity of the “tribo‐liquid” was measured by a rotary rheometer (Anton Paar MCR702).

### First‐Principles Calculations for Stress–Strain Relations

First‐principles calculations were performed using the VASP code^[^ ^73^ ^]^ and adopting the projector augmented wave method^[^ ^74^ ^]^ and local density approximation with the exchange–correlation functional of Ceperley and Alder^[^ ^75^ ^]^ as parametrized by Perdew and Zunger.^[^ ^76^ ^]^ An energy cutoff of 800 eV and a Monkhorst–Pack grid^[^ ^77^ ^]^ with a maximum spacing of 0.18 A^−1^ was adopted, achieving an energy convergence around 1 meV per atom.

The stress–strain relations under biaxial stress states that contains a shear stress and a normal compressive stress component. This approach recovers the pure shear case in the limit of zero normal stress and generally simulates indentation processes.^[^ ^62^, ^78^, ^79^ ^]^ The shape of the (deformed) unit cell and atomic relaxation were determined completely at each step by the constrained structural optimization. The starting position for each strain step was taken from the relaxed coordinates of the previous strain step to ensure the quasistatic strain path, with a strain increment of 0.01. At each step, the applied shear strain was fixed to determine the shear stress *σ_xz_*, while the other five independent components of the strain tensors and all the atoms inside the unit cell were simultaneously relaxed until the compressive stress component reaches *σ_zz_* = *c*, where *c* is a constant normal compressive stress; meanwhile, all other four components of the Hellmann–Feynman stress tensor and the force on each atom become less than 0.1 GPa and 0.005 eV A^−1^, respectively.

## Conflict of Interest

The authors declare no conflict of interest.

## Supporting information

Supporting Information

## Data Availability

The data that support the findings of this study are available from the corresponding author upon reasonable request.
