# Supplementary material for: Macroscale Robust Superlubricity on Metallic NbB2
Source: Adv Sci (Weinh). 2022 Mar 10;9(13):2103815. doi: 10.1002/advs.202103815 (PMC9069360; doi:10.1002/advs.202103815)
Supplement: Supplementary file 1 — Supporting Information [file ADVS-9-2103815-s001.pdf]

ADVANCED SCIENCE
Open Access

Supporting Information

for *Adv. Sci.*, DOI 10.1002/advs.202103815

Macroscale Robust Superlubricity on Metallic NbB_2

Jia Wang, Chang Liu, Kaifei Miao, Kan Zhang, Weitao Zheng and Changfeng Chen*

Supporting Information

Macroscale robust superlubricity on metallic NbB_2

Jia Wang, Chang Liu, Kaifei Miao, Kan Zhang, Weitao Zheng, Changfeng Chen*

Jia Wang and Chang Liu contributed equally to this work.

J. Wang, K. Miao, Prof. K. Zhang, Prof. W. Zheng
State Key Laboratory of Superhard Materials, Department of Materials Science and Key Laboratory of Automobile Materials, MOE, Jilin University, Changchun 130012, China.
Email: kanzhang@jlu.edu.cn

J. Wang
Department of Materials Science and Engineering, Jilin Jianzhu University, Changchun 130118, China.

C. Liu
International Center for Computational Methods & Software, College of Physics, Jilin University, Changchun 130012, China.

Prof. C. Chen
Department of Physics and Astronomy, University of Nevada, Las Vegas, Nevada 89154, USA.

Keywords: NbB_2, superlubricity, macroscale, superlow wear, metallic

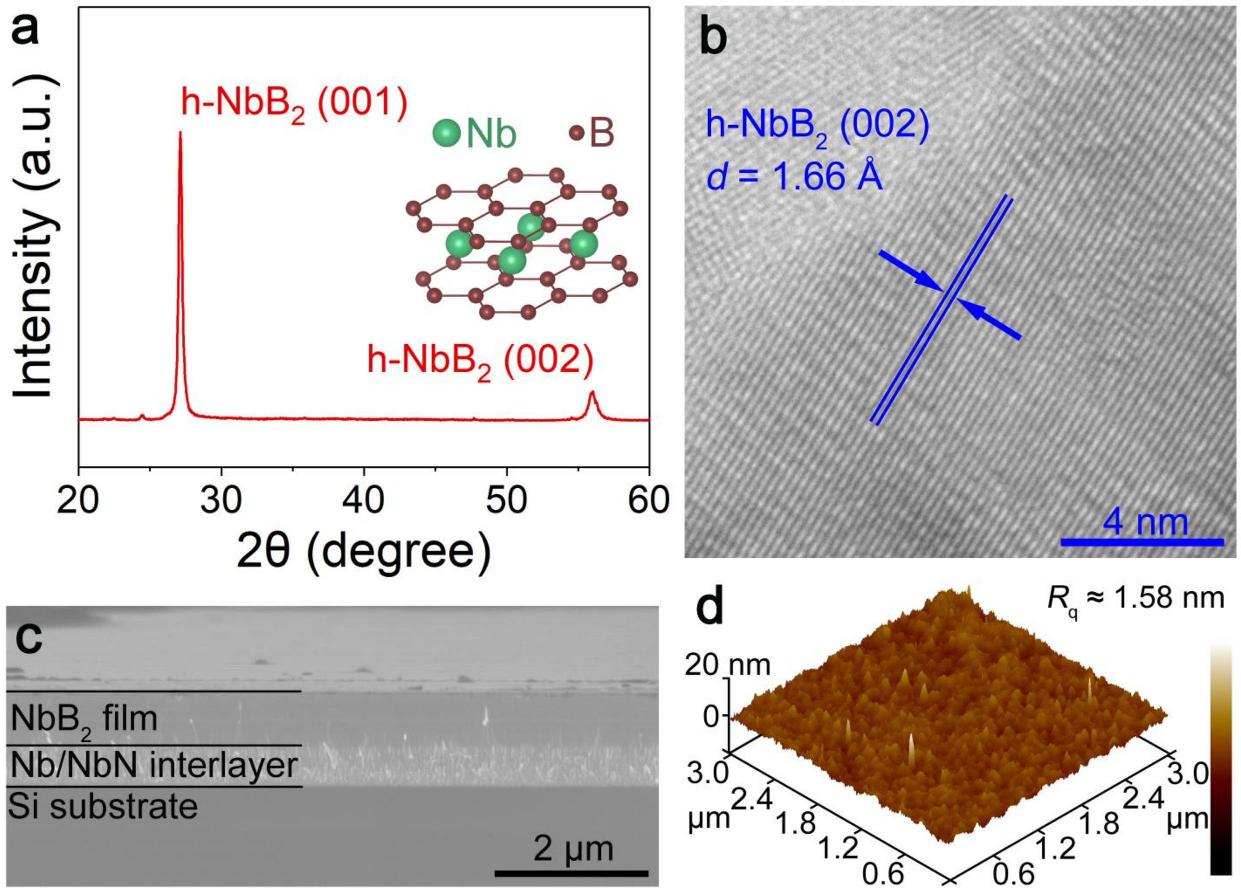

Figure S1. Structural characterization of synthesized NbB_2 film. a) X-ray diffraction (XRD) pattern. A strong peak at 27.5° accompanied by a weak peak at 56.5° are identified as originating from the (001) and (002) diffraction of the hexagonal NbB_2 structure (JCPDF: 65-0512), respectively. The clean and sharp XRD peaks indicate good crystallinity with a strong structural texture. b) An HRTEM image of the NbB_2 film. Lattice fringes with a *d* spacing of approximately 1.66 Å correspond to the (002) planes in the hexagonal NbB_2, corroborating the structural assignment from the XRD results. c) A cross-sectional SEM image of the NbB_2 film. To enhance film-substrate adhesion, an Nb/NbN interlayer about 500 nm in thickness is introduced between the film and the substrate. The thickness of the NbB_2 film is about 720 nm. d) An AFM 3D micrograph of the NbB_2 film. The root-mean-square roughness (R_q) is about 1.58 nm.

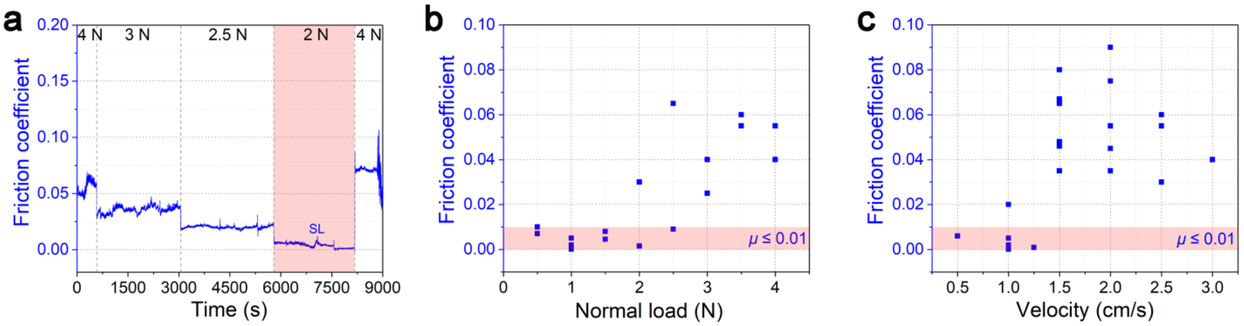

Figure S2. Friction coefficients under various applied load and velocity for the NbB_2 film in the three-phase contact environment. a) The dynamic friction curve of the NbB_2 film versus the applied load in the three-phase contact environment; b) the mean friction coefficient as a function of the load for NbB_2 film sliding against Al_2O_3 ball in the three-phase contact environment, and the data point distribution represents the results of multiple tests; the SL states indicated by the shaded bars correspond to friction coefficient (μ) below 0.01. c) The mean friction coefficient as a function of the velocity, with the SL state achieved over a range of relatively low velocity.

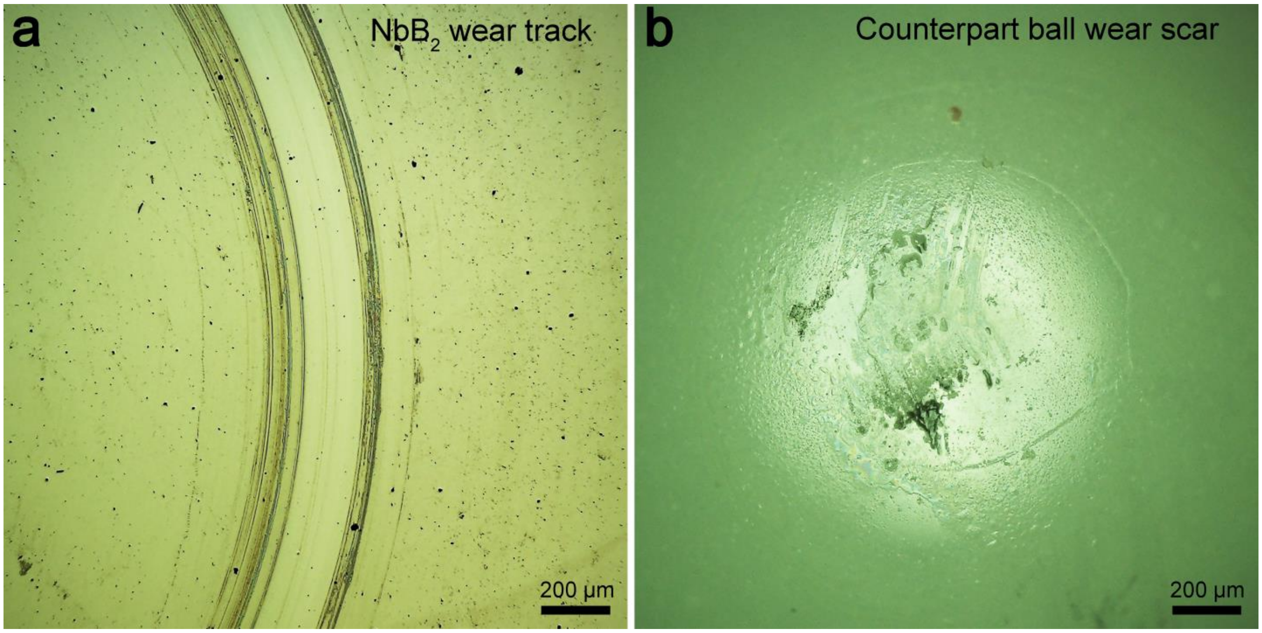

Figure S3. Wear morphology of the NbB_2 film and counterpart ball in the three-phase contact environment. a) An optical microscope image of the NbB_2 film wear track after a 10-hour tribotest. The wear track is very shallow, with some dark-colored friction products along the wear track. b) An optical microscope image of the counterpart ball wear scar after a 10-hour tribotest. Dark friction products are attached to the center of the wear scar, while the edge of the wear scar is clean.

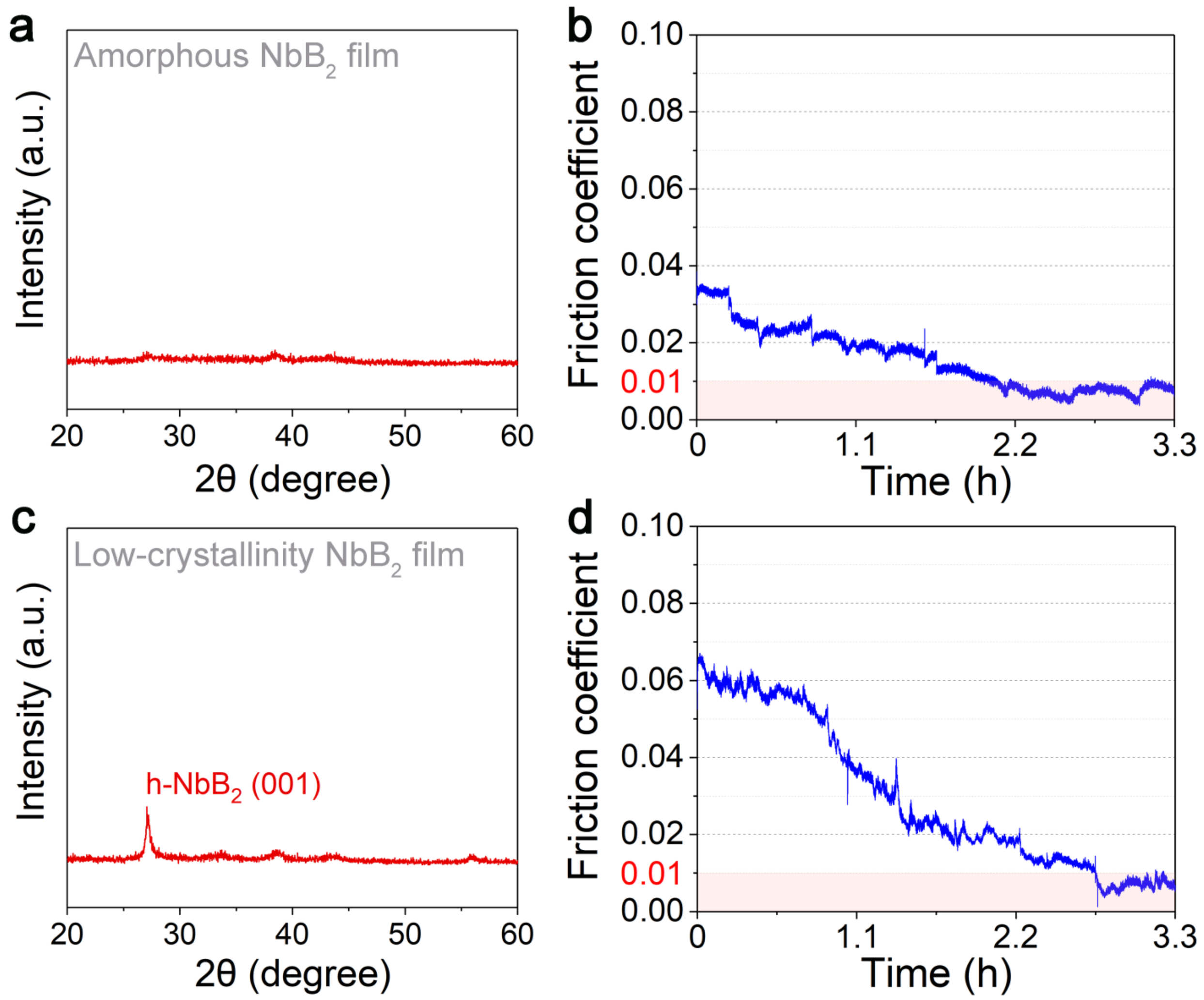

Figure S4. Tribotests of NbB_2 films in less crystallized forms. a) The XRD pattern of an NbB_2 film in typical amorphous structure. b) Dynamic friction coefficient of the amorphous NbB_2 film in the three-phase contact mode. After a break-in period around 2.0 hours, the friction coefficient drops below 0.01, entering an SL state that lasts for the subsequent test period with an average friction coefficient of 0.0076. c) The XRD pattern of an NbB_2 film with low crystallinity compared to that shown in Figure S1a. d) Dynamic friction coefficient of the low-crystallinity NbB_2 film in the three-phase contact mode. After a break-in period of about 2.8 hours, the film enters an SL state with an average friction coefficient of 0.0069.

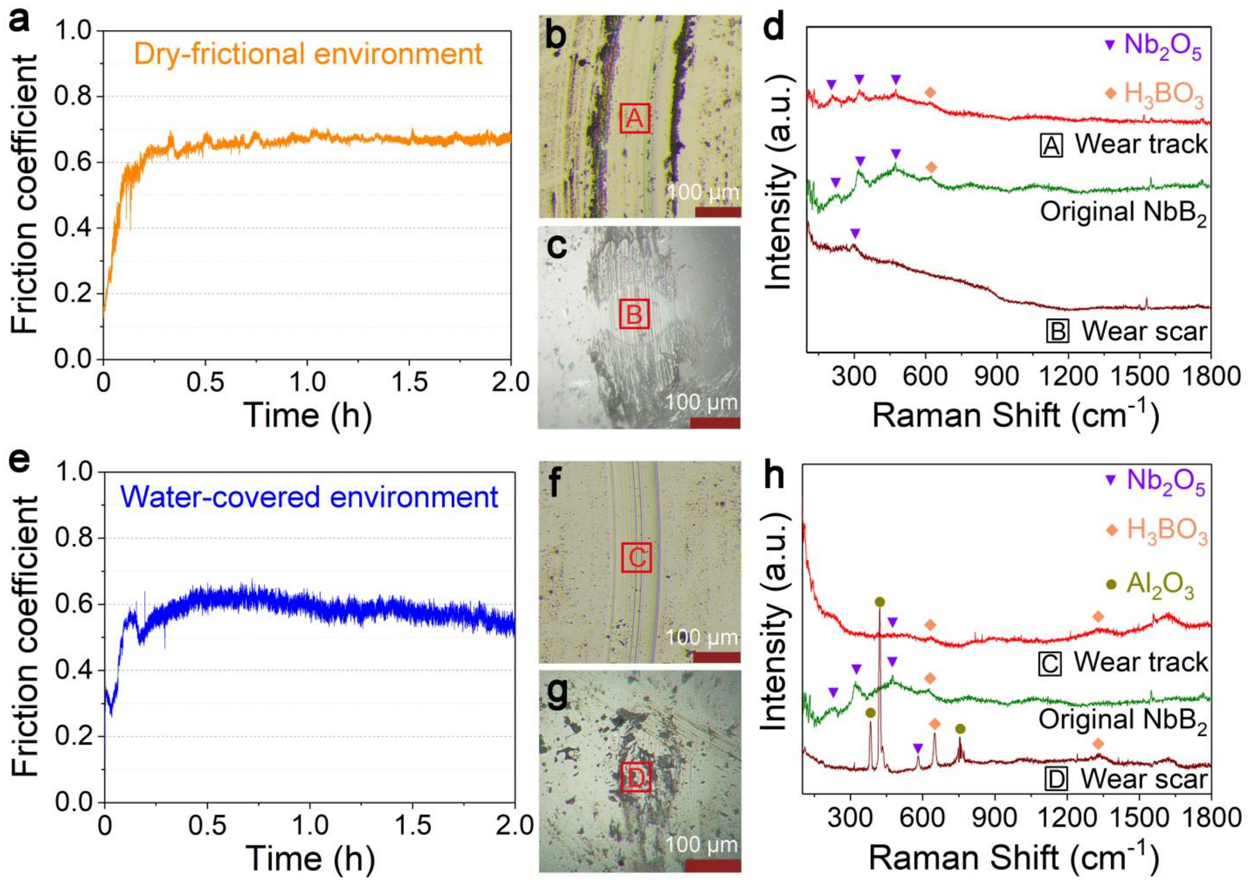

Figure S5. Tribological behaviors of NbB_2 films in dry or fully water-covered frictional environments. a) Dynamic friction coefficient of the NbB_2 film in good crystallinity measured in a dry frictional environment. The mean friction coefficient of the NbB_2 film during the stabilization period is approximately 0.66. Optical microscope images of (b) the film wear track and (c) the counterpart ball wear scar, both revealing notable abrasions. A significant accumulation of wear debris accompanied by some degree of adhesion is seen on the edge of the wear track and wear scar. d) Raman spectra of the wear track, original NbB_2 film and wear scar in the dry frictional environment. A comparison of the Raman signals from the wear track and the wear scar after the tribotest finds no significant changes at the wear track center, indicating that the wear track maintains an oxidized state during the friction process. e) Dynamic friction

coefficient of the NbB_2 film in good crystallinity measured in a fully water-covered frictional environment. The mean friction coefficient of the NbB_2 film during the stabilization period is approximately 0.59. Optical microscope images of (f) the film wear track and (g) the counterpart ball wear scar, showing no wear debris attached on the wear track, likely caused by the scouring effect of water. h) Raman spectra of the wear track, original NbB_2 film and wear scar in the fully water-covered frictional environment. Raman spectra taken after the tribotest show reduction of signals belonging to Nb_2O_5 and H_3BO_3 at the center of the wear track and wear scar compared to those of the original NbB_2 film, indicating that under the influence of an ample amount of water, few tribo-products remain at the frictional interface.

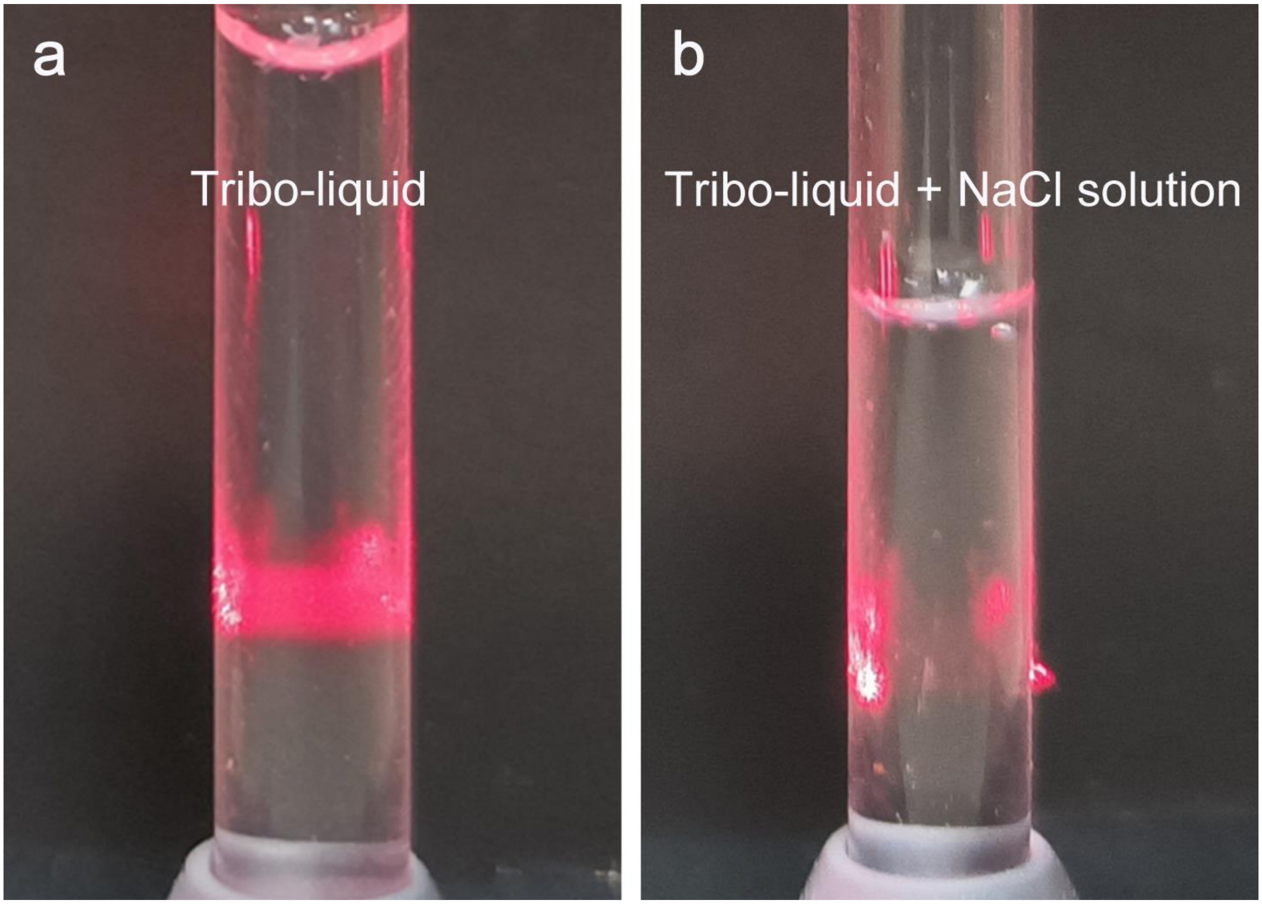

Figure S6. Tyndall effect of the tribo-liquid collected after the tribotest in the three-phase contact environment. a) When the tribo-liquid is irradiated directly under an infrared light, it exhibits a red optical pathway, showcasing the classic Tyndall effect indicating that a certain concentration of charged colloids exists in the tribo-liquid.[1] b) When an electrolyte solution (NaCl) is injected into the tribo-liquid, the discernible beam path disappears, indicating that the colloidal stability of an electrostatically stabilized dispersion has been broken down.

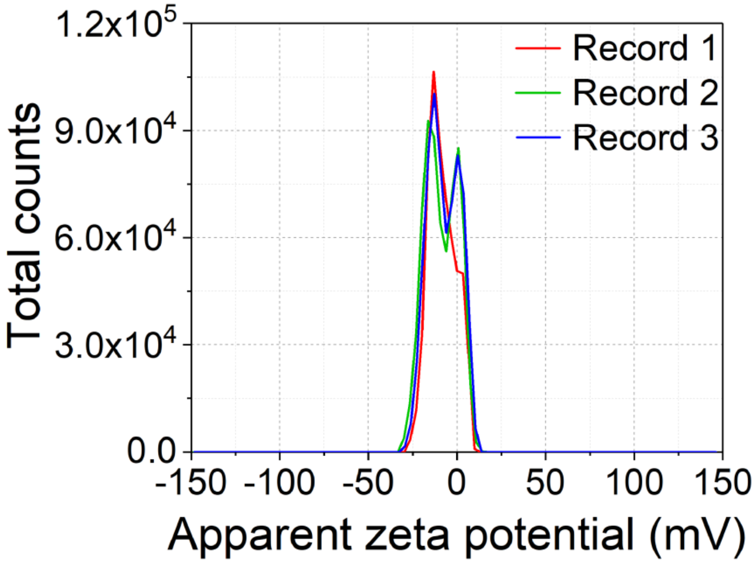

Figure S7. Zeta potential distributions of the tribo-liquid. The zeta potential distributions of the tribo-liquids collected after the tribotests; the results shift to negative values with an average of -8.45 mV.

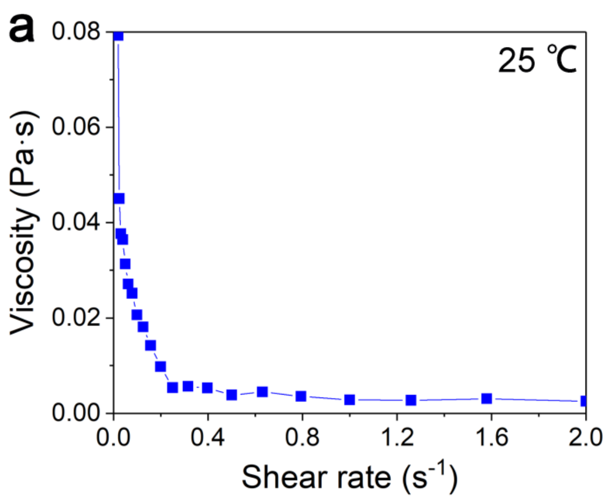

Figure S8. Effects of shear rate and temperature on the viscosity of the tribo-liquid. a) Viscosity of the tribo-liquid at 25 °C as a function of shear rate. When the shear rate is 0.796 s^{-1} (corresponding to the velocity of 1 cm/s), the viscosity of the tribo-liquid remains at 0.0036 Pa·s. b) Viscosity of the tribo-liquid at the shear rate of 0.796 s^{-1} as a function of temperature. Viscosity decreases with rising temperature.

To clarify the lubrication regime during the SL period, the hydration layer thickness (h_c) between the tribopairs and lambda-ratio (λ) have been calculated,[2] as follows:

The minimum h_c was calculated by the Hamrock-Dowson formula:

$$h_c = (2.69G^{0.53}RU^{0.67}/W^{0.067})\cdot(1\text{-}0.61e^{-0.73k}) \qquad (1)$$

where

$$G = \alpha E' \qquad (2)$$

$$U = \eta u/E'R \qquad (3)$$

$$W = F/E'R^2 \qquad (4)$$

$$R = E''d^3/6F \qquad (5)$$

$$E' = 2/[(1\text{-}\upsilon_1^{\,2})/E_1+(1\text{-}\upsilon_2^{\,2})/E_2] \qquad (6)$$

$$E'' = 1/[(1-\upsilon_1^2)/E_1+(1-\upsilon_2^2)/E_2] \qquad (7)$$

Here k is a coefficient (approximately 1) and α refers to pressure-viscosity coefficient, which is usually in the range of 4~8 GPa^{-1} for water-based lubricants.[3] The viscosity (η) of the tribo-liquid is 0.0036 Pa·s. The applied load (F) and velocity (u) are 1 N and 0.01 m/s, respectively. E_1 and E_2 denote the elasticity moduli of the NbB_2 film and Al_2O_3 ball and υ_1 and υ_2 denote the respective Poisson's ratios. E_1 and E_2 are approximately 367.6 and 350 GPa, respectively, and υ_1 and υ_2 are about 0.75 and 0.22, respectively. E' and E'' are the equivalent elastic modulus and effective elastic modulus of the tribopairs, respectively. R is the effective ball radius and d represents the wear scar diameter on the counterpart ball (approximately 0.0001 m). The value of h_c is 50.65 nm under the SL state, indicating the existence of the fluid layer between the tribopairs. The lubrication regime was defined by λ:

$$\lambda = h_c/(\sigma_1^2+\sigma_2^2)^{0.5} \qquad (8)$$

where σ_1 and σ_2 are the surface roughness of the wear area on the film (approximately 9 nm) and ball (approximately 14 nm), respectively. The value of λ is about 3.05 ($\geq$ 3), indicating that the lubrication regime is located in hydrodynamic lubrication.

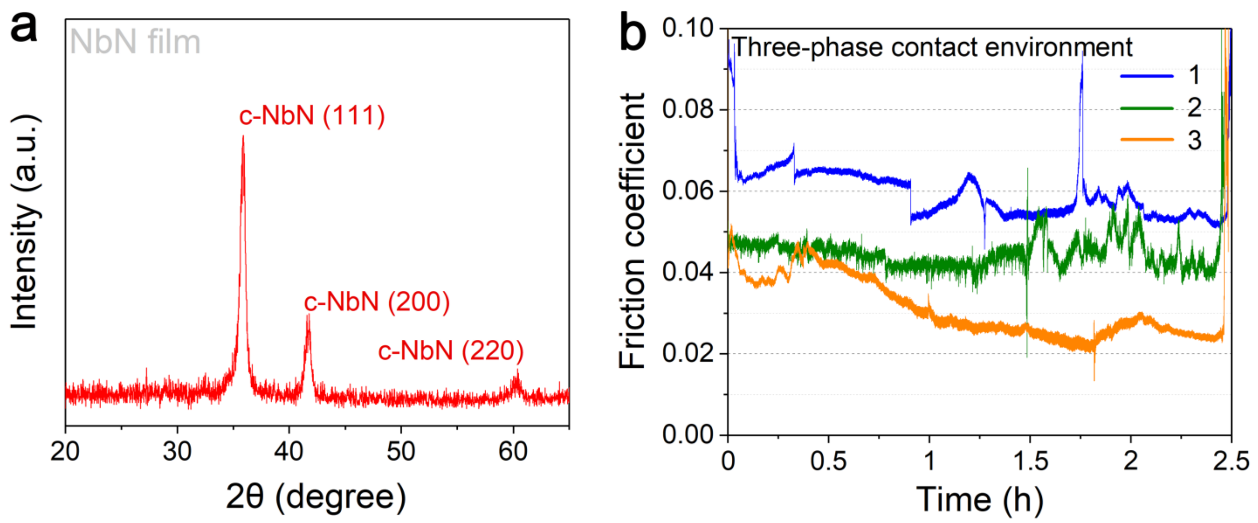

Figure S9. Tribotests on an NbN film in the three-phase contact environment. a) The XRD pattern of the NbN film. Three peaks at 35.7°, 41.3° and 60.1° are identified as originating from the (111), (200) and (220) diffractions of the cubic NbN structure (JCPDF: 65-9399), respectively. b) Dynamic friction coefficients of the NbN film. Three repeated tests were performed and the corresponding results indicate that the average value is maintained between 0.033 and 0.059 in the three-phase contact environment, but the film did not achieve the SL state. When the friction tests exceeded about 2.5 hours, the observed film failure occurs.

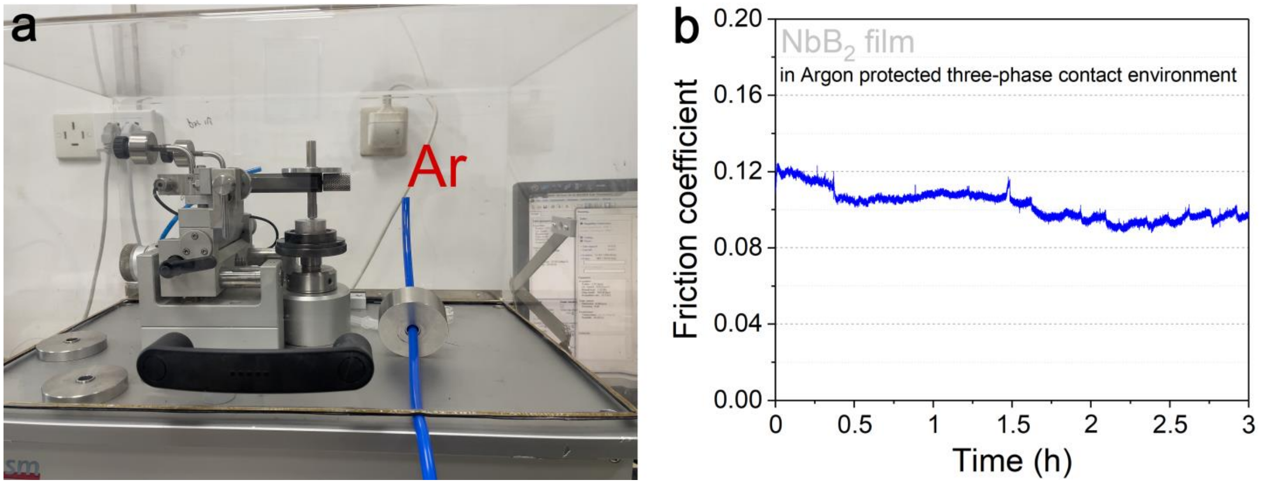

Figure S10. The tribotest on the NbB_2 film in the argon protected three-phase contact environment. a) The image of the ball-on-disk tribotester with argon protection. Argon is continuously pumped into the tribotest chamber near the tribopairs to isolate oxygen. During the friction process, a film-water-argon three-phase contact mode is formed between the tribopairs. b) Dynamic friction coefficient of the NbB_2 film. In this argon based three-phase contact environment, the NbB_2 film cannot achieve the SL state because the tribopairs are in poor contact with oxygen. The average friction coefficient of the NbB_2 film in the argon environment is about 0.103, which is 13.7 times larger than that in the ambient air environment.

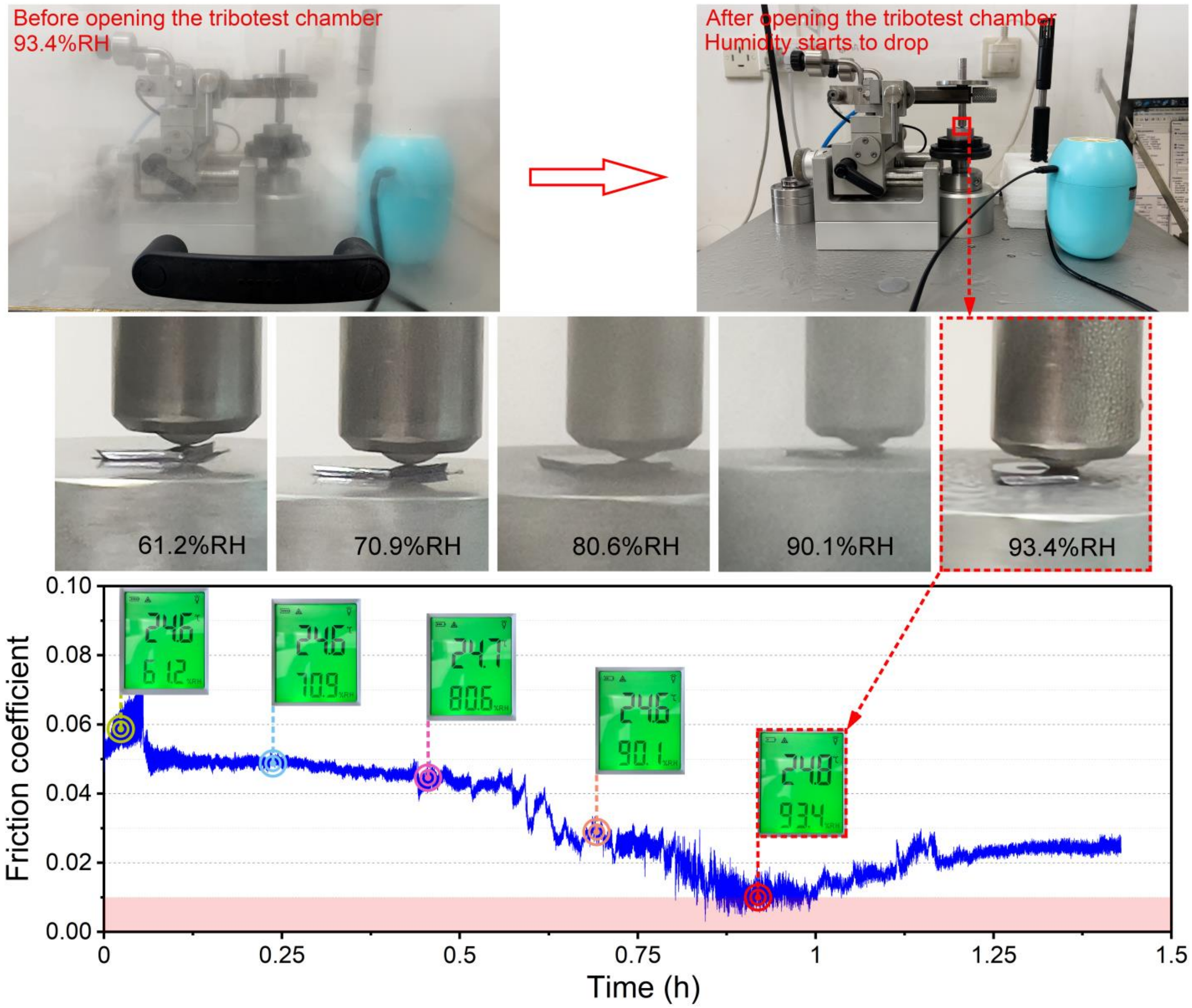

Figure S11. Tribotests on the NbB_2 film in a controlled humidity environment. The friction coefficient of the NbB_2 film decreases with the increasing humidity inside the chamber. When humidity exceeds 90.1%, the large amount of water vapor leads to low visibility inside the chamber, and the friction coefficient fluctuates greatly but still decreases. When the humidity reaches about 93.4%, the moisture from the humid environment gathers on the film surface and covers the contact area between the tribopairs, forming the same type of "water bridge" achieved by directly adding a water droplet between the tribopairs, and the NbB_2 film enters the SL state. This result shows that a steady-state three-phase contact can be realized naturally at the dynamic

friction interface in a high-humidity environment. Moreover, as long as it is in a three-phase contact environment, the SL state of the NbB_2 film can be maintained. After opening the chamber, with the water evaporation between the tribopairs, the friction coefficient of the NbB_2 film increases gradually and the SL state is lost.

References

[1] D. Li, M. B. Muller, S. Gilje, R. B. Kaner, G. G. Wallace, *Nat. Nanotechnol.* **2008**, 3, 101.
[2] G. Tang, Z. Wu, F. Su, H. Wang, X. Xu, Q. Li, G. Ma, P. K. Chu, *Nano Lett.* **2021**, 21, 5308.
[3] T. Han, C. Zhang, J. Li, S. Yuan, X. Chen, J. Zhang, J. Luo, *J. Phys. Chem. Lett.* **2020**, 11, 184.
